# Biosynthesis of Lactobionic Acid in Whey-Containing Medium by Microencapsulated and Free Bacteria of *Pseudomonas taetrolens*

**DOI:** 10.1007/s12088-021-00944-4

**Published:** 2021-05-11

**Authors:** Kamila Goderska

**Affiliations:** Department of Food Technology of Plant Origin, Department of Fermentation and Biosynthesis, Faculty of Food Science and Nutrition, Poznan University of Life Sciences, Wojska Polskiego 31, 60-624 Poznan, Poland

**Keywords:** Lactobionic acid, *Pseudomonas taetrolens*, Whey

## Abstract

The aim of this research was to develop a method of its production from whey using bacteria of the species *Pseudomonas taetrolens*. Analyses of the lactobionic acid production method from whey showed that the following factors have a significant effect on its efficiency: the frequency of whey batch feeding, pH and the type of bacteria application, i.e. microencapsulated vs. free. Lactose and lactobionic acid were assayed using high performance liquid chromatography (HPLC) and liquid chromatography-electrospray ionization-tandem mass spectrometry (LC–ESI–MS). The highest concentration of lactobionic acid of 22.03 mg/cm^3^ was obtained when whey was batch fed at 72-h intervals, pH was maintained at 6.25 and bacteria were enclosed in alginate microcapsules.

**Supplementary Information:**

The online version contains supplementary material available at 10.1007/s12088-021-00944-4.

## Introduction

Lactobionic acid is a polyhydroxy acid (PHA) composed of a molecule of galactose (a chemically neutral sugar) and a molecule of gluconic acid (naturally found in the skin and capable of retaining considerable amounts of water), containing numerous hydroxy groups. Its systematic name is 4-O-β-D galactopyranosylo-D-gluconic acid [1]. It is a weak acid with a molecular mass of 358.3 Da, a sweet taste and energy value of 2 kcal/g [2, 3]. Lactobionic acid is a substance of poorly known structure and limited applications due to high costs of its production [4]. For this reason microbiological production of this acid with possibly the highest efficiency and requiring no enzyme purification seems to be an interesting alternative and a cutting-edge technology based on the latest advances and dedicated processes of biotechnology [5–10]. The first studies concerning the production of lactobionic acid were conducted using strains of *Zymommonas mobilis* and genetically engineered strains of *Escherichia coli.* Results were presented in a publication: Goderska K., Juzwa W., Szwengiel A., Czarnecki Z. Lactobionic acid production by glucose-fructose oxidoreductase from *Zymomonas mobilis* expressed in *Escherichia coli*. Biotechnology Letters 2015, 37(10): 2047–2053 [11]. Due to the very limited number of reports concerning the production of lactobionic acid using *Pseudomonas taetrolens* experiments were conducted on the fermentation of whey using a strain of this microbial species. Results were presented in a publication: Goderska K., Szwengiel A., Czarnecki Z.; The utilization of *Pseudomonas taetrolens* to produce lactobionic acid. Applied Biochemistry and Biotechnology 2014, 173, 2189–2197 [12].

In the literature we can find the publication about biowaste materials for production new bioproducts and various immobilization methods for biotransformation applications.

Environmental pollution resulting from inadequate waste management and continous emission of greenhouse gases (GHGs) has encouraged research on the development of suitable strategies for remediating such adverse effects on the environment. Both methane (CH_4_) and carbon dioxide (CO_2_) are major greenhouse gases (GHGs); hence, effective processes are required for their conversion into useful products. CH_4_ is used by a few groups of methanotrophs to produce methanol. However, to achieve economical and sustainable CH_4_ reduction strategies, additional strains are needed that can exploit natural CH_4_ feed stocks. In the study of Patel et al. (2016) [13], they evaluated methanol production by *Methylosinus sporium* from CH_4_ and synthetic gas.

In the study of Patel et al. (2021) [14], potato peels were subjected to anaerobic digestion (AD) to produce biogas (methane [CH_4_] and carbon dioxide), which was subsequently used as a substrate for methanol production by methanotrophs. This is the first study on immobilization of methanotrophs on banana leaves for producing methanol from potato peels AD-derived biogas. The study of Patel et al. (2020) [15] aimed to establish a unique approach for the production of methanol from methane (CH_4_) in the presence of paraffin oil mediated by methanotrophs immobilized on coconut coir (CC). In the next study of Patel et al. (2020) [16], chitosan modified with glutaraldehyde (GLA), 3-aminopropyltriethoxysilane (APTES), polyethyleneimine, and APTES followed by GLA (APTES-GLA) as a support material was used to improve methanol production from biogas. This is the first report on the immobilization of methanotrophs on chemically modified chitosans to improve cell loading and relative efficiency, and its potential applications in the conversion of greenhouse gases to methanol.

Raw biogas can be an alternative feedstock to pure methane (CH_4_) for methanol production. In the investigation of Patel et al. (2016) [17], they evaluated the methanol production potential of *Methylosinus sporium* from raw biogas originated from an anaerobic digester.

Biohythane may be used as an alternative feed for methanol production instead of costly pure methane. In the study of Patel et al. (2017) [18], methanol production potential of *Methylocella tundrae* immobilized through covalent immobilization, adsorption, and encapsulation was evaluated. In the study of Patel et al. (2018) [19], co-cultures of the methanotrophs *Methylocella tundrae*, *Methyloferula stellata*, and *Methylomonas methanica* were evaluated for improving methanol production with their application. This is the first report of methanol production from defined free and immobilized co-cultures using simulated biogas mixtures as feed. This investigation of Patel et al. (2020) [20] demonstrated that the methanotrophs based coculture approach improves the methanol production from CH_4_. Co-culture consisting of *M. bryophila* and *M. stellate* showed better methanol production over their pure cultures. The immobilization of co-cultures within PVA retained high RE for methanol production and led to better stability over free cells. In the study of Patel et al. (2020) [21] Type I (*Methylomicrobium album*) and II (*Methyloferula stellata*) methanotrophs were encapsulated by alginate and polyvinyl alcohol (PVA) to improve methanol production from simulated biogas [methane (CH_4_) and carbon dioxide (CO_2_)] in the presence of CH_4_ vector.

Utilizing this freely available waste-water along with biodiesel industry waste- crude glycerol for bio-hydrogen production is being reported by Prakash et al. (2018) [22]. The study of Kumar et al. (2019) [23] details a potential approach to producing biofuels from agricultural biomass, the applicability of which can be improved through up‐scaling. In the present study, an immobilized enzyme cocktail containing laccase was isevaluated in regard to its ability to enhance the saccharification and fermentation processes by reducing the amount of phenolic compounds produced.

Biodiesel manufacturing units discharge effluents rich in glycerol. The need is to convert crude glicerol (CG) into useful products such as hydrogen (H_2_). In the study of Kumar et al. (2015) [24]*, B. thuringiensis* could transform CG, on limited resources – minimal medium with sodium nitrate, by immobilizing them on cheap and easily available biowaste, which makes it a suitable candidate for H_2_ production on a large scale.

In the study of Patel et al. (2016) [25], an integrative approach to produce biohydrogen (H_2_) and polyhydroxyalkanoates (PHA) from the wastes of biological origin was investigated. Co-digestion of biowastes for hydrogen (H_2_) production using defined mixed cultures can overcome the high risk of failure due to contamination and imbalanced nutrient status. H_2_ production from biowastes—pea-shells, potato peels (PP), onion peels (OP) and apple pomace, either individually or in various combinations was evaluated by hydrolyzing with defined hydrolytic mixed bacterial culture (MHC5) and subjecting the hydrolysate to mixture of defined H_2_ producers (MMC6) [26]. Further economic improvement in this process can be achieved through its integration with processes leading to the production of CH_4_, PHA or biomethanol, through biorefinery approach.

The aim of this study was to indicate new directions for the utilisation of lactobionic acid as well as present the dependence between environmental conditions and the efficiency of microbiological production of lactobionic acid using bacteria *Pseudomonas taetrolens* DSM 21104, which may contribute to future applicatory solutions. Due to the very limited number of publications on this subject this study contributes to research on the production of lactobionic acid ensuring the highest possible efficiency, while indication of new directions for its application provides an additional value for pure science. In view of the above, the following research hypothes was proposed:

Application of controlled environmental conditions and immobilisation of microorganisms during culture of bacteria *Pseudomonas taetrolens* may intensify the production of lactobionic acid in the substrate containing whey.

Verification of individual hypotheses included:

Analyses of the effect of culture methods of bacteria *Pseudomonas taetrolens* and such parameters as pH or microbial forms on the on the efficiency of lactobionic acid production. Conversion rate of lactose contained in whey to lactobionic acid, the use of lactose in batch and fed-batch processes.

Obtaining the highest possible yield of lactobionic acid might provide an alternative to the production of this acid by biocatalytic production of lactobionic acid via enzymatic synthesis including enzyme purification.

## Materials and Methods

### Inoculum Preparation

A loopful of *P. taetrolens* from a fresh Tryptone Soya Broth agar plate was used to inoculate a 500 mL Erlenmeyer flask containing 100 mL of Tryptone Soya Broth medium. This flask was incubated on an orbital shaker at 250 rpm and 30 °C for 24 h. Actively growing cells from this culture were then employed as inoculum for the production of lactobionic acid in bioreactor seed cultures containing sweet whey, as subsequently reported.

### Immobilisation of Bacteria by Encapsulation with a Liquid-Core

Bacteria encapsulation was conducted using the method of extrusion as described by Goderska and Czarnecki (2008) [27] Fig. S1.

### Preparative Scale Batch Reactions

Batch cultivations were performed in a 2 l bioreactor (Biostat Braun Biotech International, Germany) with 1 l of whey (2% lactose) as working volume at 30 °C. An inoculum level of 10% (v/v) was used. An efficient two-stage pH-shifted bioconversion strategy was adopted as previously described [14]: pH was controlled above 6.25 (pH was left uncontrolled above this value during the growth phase and subsequently maintained at 6.25) by means of computer-controlled peristaltic pumps via automatic addition of 2 M NaOH. These prior conditions were applied to all cultivations unless otherwise specified. Cultivations were carried out in duplicate as independent experiments.

### Whey Preparation

Cheese rennet whey (Poznan, Poland) was diluted with distilled water (1:1 v/v) and adjusted to pH 6.25 with 6 M NaOH prior to sterilization using a tangential microfiltration device equipped with a PVDF membrane-cassette of 0.22 μm pore size (Millipore, Massachusetts, USA).

### Microbial Production of Lactobionic Acid in a Bioreactor Using *Pseudomonas Taetrolens* in the Free and Encapsulated Form

Bioconversion of lactose from whey to lactobionic acid was run in a BIOSTAT®B fermenter (B. Braun Biotech International, Germany) equipped with a fermenter tank of 2 dm^3^, a heating jacket, a mixer, a pH electrode, a thermometer, an air inlet filter, a rotameter, an oxygen gas electrode and metering pumps proportioning the acid and a base or medium. The volume of 1000 cm^3^ liquid medium (whey) was transferred to a 2 dm^3^ pre-sterilised bioreactor and sterilised. Next the culture of *Pseudomonas taetrolens* (2 × 50 cm^3^) was centrifuged and the supernatant was decanted. The precipitate was supplemented with saline and the medium was inoculated in the bioreactor using a microbial solution (inoculum). Incubation was run at a temperature of 30 °C at 120 rpm and continuous aeration of the culture (0.5 dm^3^/min). Samples (10 cm^3^) were collected at 24-h intervals. The pH value was adjusted using 1 M HCl as well as 1 M and 2 M NaOH. Constant parameters, which were adopted in all cultures were as follows: temperature—30 °C, aeration – 0.5 dm^3^/min, mixing—120 rpm. Variable parameters in the process of lactobionic acid production included: time, after which liquid culture medium was collected and sterile whey was supplemented, the form of microorganisms – free vs. immobilised, and ambient pH.

Variants of culture:

- Cultures 1 and 2: free bacteria, liquid culture medium (400 cm^3^) collected at every 24 h and supplemented with sterile whey (500 cm^3^), no pH adjustment;
- Cultures 3 and 4: free bacteria, liquid culture medium (400 cm^3^) collected at every 72 h and supplemented with sterile whey (500 cm^3^), no pH adjustment;
- Culture 5: immobilised bacteria, liquid culture medium (400 cm^3^) collected at every 72 h and supplemented with sterile whey (500 cm^3^), no pH adjustment;
- Culture 6: free bacteria, liquid culture medium (400 cm^3^) collected at every 72 h and supplemented with sterile whey (500 cm^3^), constant pH = 6.25;
- Culture 7: immobilised bacteria, liquid culture medium (500 cm^3^) collected at every 72 h and supplemented with sterile whey (500 cm^3^), constant pH = 6.25.

Changes in contents of lactose and lactobionic acid were determined using high performance liquid chromatography (HPLC) and LC–ESI–MS analyses.

### Analysis of Lactose and Lactobionic Acid

Lactose and lactobionic acid were measured by HPLC using Rezex ROA Organic Acid column (300 × 7.8 mm; Phenomenex International, Torrance, CA, USA) with detection at 210 nm- RI detector and PAD detector with an eluent of 0.025 M sulfuric acid at 0.5 ml min−1. The compounds present in each sample were identified by comparing their retention times with those of standards. The chromatographic co-elution of lactobionic acid (LA) and lactose (L) require the simultaneous use refractometric detector (RI) and photodiode array (PDA) detector. The calibration curves for LA (using RI and PDA detectors) and L (using RI detector) were done. The concentration of LA (x1) was calculated directly from PDA at 210 nm (y1 = 1.451*105x1, R^2^ = 0.9992, where y1 = peak area of LA). The refractometric signal (y2) equivalent to the signal PDA (y1) for LA was calculated using formula y2 = 1.450*105x1, R^2^ = 0.9998. The concentration of lactose (x3) was calculated indirectly with RI detector (y3—y2 = 2,79*105x3, R^2^ = 0.9999, where y2 = peak area of LA co-eluted with L).

## Results and Discussion

The first run culture involved oxidation of lactose from whey in a bioreactor by free bacteria *Pseudomonas taetrolens*. Samples were collected at every 24 h, at the same time collecting liquid culture medium and supplementing it with sterile whey. The culture was run using free microorganisms at continuous aeration (0.5 dm^3^/min) and thermostatic control (30 °C), with no adjustment of pH. The highest concentration of lactobionic acid (24.75 mg/cm^3^) was obtained at 48 h of culture. At the successive hours of culture the concentration of lactobionic acid, being a product of lactose bioconversion, decreased to 3.89 mg/cm^3^. At 264 h of culture the concentration of lactobionic acid increased gradually, while at 312 h culture it was 8.53 mg/cm^3^ Fig. S2.

The second culture variant involved oxidation of lactose from whey in a bioreactor – also by free bacteria *Pseudomonas taetrolens*, which was a replication of culture 1, maintaining the same conditions. The concentration of lactobionic acid increased gradually to 120 h of culture and amounted to 18.79 mg/cm^3^. Afterwards the concentration of lactobionic acid was decreasing up to 264 h, at which an increase in the concentration of the acid was recorded, followed by its reduction until the last hours of culture. In culture 2 the concentration of produced lactobionic acid was lower than in culture 1, but based on the recorded results the collection of the medium from the bioreactor and its replacement with new medium was extended in further experiments to 72 h. Lactose was found in whey up to 72 h of culture and for this reason a new batch of whey was added after that time Fig. S3.

The third culture involved oxidation of lactose from whey in a bioreactor by free bacteria *Pseudomonas taetrolens* at an extended supplementation time of fresh whey. The culture was run using free microorganisms at continuous aeration (0.5 dm^3^/min), thermostating (30 °C) and no pH adjustment applied.

The highest concentration of lactobionic acid was obtained at 96 h of culture and it amounted to 16.32 mg/cm^3^. Liquid culture medium was collected at 120, 216 and 312 h and supplemented with sterile whey. After supplementation of culture with fresh medium the concentration of lactobionic acid increased slightly to 7.23 mg/cm^3^, 2.22 mg/cm^3^ and 1.3 mg/cm^3^ Fig. S4.

The fourth culture involved oxidation of lactose from whey in a bioreactor by free bacteria *Pseudomonas taetrolens* with an extended supplementation of fresh whey, which was a replication of culture 3, maintaining the same conditions. The concentration of lactobionic acid in the culture was gradually increasing, with its highest concentration (8.54 mg/cm^3^) recorded at 96 h. After the collection of the liquid culture medium at 120, 216 and 312 h the concentration of the acid was observed to decrease, to gradually increase again after supplementation with fresh medium. The concentrations of produced lactobionic acid were markedly higher than in culture 3 for 336 h of cultures, whereas higher concentrations of produced lactobionic acid were found only up to 120 h in culture 3 Fig. S5.

In view of the promising preliminary results concerning the production of lactobionic acid using microencapsulated bacteria *Pseudomonas taetrolens* the successive stages of the study involved fermentation with immobilised microorganisms. The results were compared with those of culture, in which bacteria in the free form were used.

The fifth culture variant involved oxidation of lactose from whey in a bioreactor by bacteria *Pseudomonas taetrolens* following their encapsulation. The culture was run using encapsulated microorganisms at continuous aeration (0.5 dm^3^/min), maintaining constant temperature of culture (30 °C) with no adjustment of pH. Following collection of liquid culture medium at 72-h intervals the concentration of the acid was observed to decrease, to increase gradually following fresh medium supplementation. The highest concentration of lactobionic acid, amounting to 3.99 mg/cm^3^, was obtained at 144 h of culture Fig. S6.

The sixth culture variant involved oxidation of lactose from whey in a bioreactor with constant ambient pH by free bacteria *Pseudomonas taetrolens*. The culture was run using free microorganisms at continuous aeration (0.5 dm^3^/min), maintaining constant temperature of the culture (30 °C) and adjusting pH to 6.25. Following collection of liquid culture medium at every 72 h the concentration of the acid decreased, to gradually increase again after supplementation with fresh medium. The highest concentration of lactobionic acid of 1.47 mg/cm^3^ was obtained at 216 h culture Fig. S7.

The seventh culture variant involved oxidation of lactose from whey in a bioreactor with constant ambient pH by encapsulated bacteria *Pseudomonas taetrolens*. The culture was run using encapsulated microorganisms at continuous aeration (0.5 dm^3^/min), maintaining constant temperature of the culture (30 °C) and adjusting pH to 6.25. Following collection of liquid culture medium at 72-h intervals the concentration of the acid decreased, to gradually increase again after supplementation with fresh medium. The highest concentration of lactobionic acid was obtained at 24 h of culture and it amounted to 22.03 mg/cm^3^ Fig. S8.

Cultures No. 1–7 were prepared to find whether the frequency of fresh whey dosage, its pH and the immobilisation of bacteria affected the efficiency of lactobionic acid biosynthesis. The results of selected cultures were analysed. Changes in the concentration of lactobionic acid in cultures No. 1 and No. 4 were compared to confirm or reject the hypothesis assuming the influence of fresh medium dosage frequency on the acid biosynthesis efficiency. Cochran’s, Hartley’s and Bartlett’s tests at a significance level of α = 0.05 were used to check at every hour the homogeneity of variance in the lactobionic acid concentration in both cultures, which differed only in the fresh medium (whey) dosage frequency. All variances were homogeneous. Next, the hypothesis of the equality of averages was rejected after a classic analysis of variance as it indicated significant differences between them at specific times of culturing. Culture No. 1 was characterised by higher yield of lactobionic acid than culture No. 4. The statistical analysis showed that the change of the substrate and its replacement frequency significantly influenced the lactobionic acid production efficiency.

The analysis of changes in the concentration of lactobionic acid in cultures No. 4 and 5 was supposed to show whether the type of bacteria used in the research (free or encapsulated bacteria) differentiated the LBA yield at unregulated pH. There were free bacteria in culture No. 4 and microencapsulated bacteria in culture No. 5. The homogeneity of variance at individual culturing times was checked by means of Cochran’s, Hartley’s, and Bartlett’s tests at a significance level α = 0.05. All the variances were homogeneous. Next, the hypothesis of the equality of averages was rejected after a classic analysis of variance as it indicated significant differences between them. Culture No. 4 was characterised by higher yield of lactobionic acid than culture No. 5. The statistical analysis showed that the encapsulation of bacteria significantly influenced the lactobionic acid production efficiency at unregulated pH.

Changes in the concentration of lactobionic acid in cultures No. 6 and 7 were analysed to find whether the immobilisation of encapsulated bacteria affected the LBA efficiency at controlled pH. There were free bacteria in culture No. 6 and encapsulated bacteria in culture No. 7. A steady pH of 6.25 was maintained in both cultures. The homogeneity of variance at individual culturing times was checked by means of Cochran’s, Hartley’s, and Bartlett’s tests at a significance level α = 0.05. All the variances were homogeneous. Next, the hypothesis of the equality of averages was rejected after a classic analysis of variance as it indicated significant differences between them. The analyses proved that at regulated pH the encapsulated bacteria were also more efficient than free bacteria in lactobionic acid production.

The research results also solved another problem – pH regulation helps to maximise the LBA biosynthesis in the presence of whey. In our study pH was maintained at 6.25. We were guided by the results of the research by Alonso et al. (2013), who recommended maintaining this parameter at 6.5. They explained that these conditions guaranteed the highest metabolic activity of the cells. It is possible that the highest LBA efficiency was observed due to the constant pH (6.25) in culture No. 7.

In my opinion, the encapsulation of bacteria was another reason for the success of culture No. 7. This conclusion comes from the comparison of the results of cultures No. 6 and No. 7. There were free bacteria in culture No. 6, whereas culture No. 7 was based on immobilised bacteria. Alginate capsules may have provided protection against *P. taetrolens*. However, on the other hand, there were also encapsulated microorganisms in culture No. 5, but without pH regulation. It resulted in a lower yield of lactobionic acid. Therefore, we can conclude that alginate capsules have a protective function and increase the production efficiency of lactobionic acid at pH of 6.25.

It is known that some factors may jointly inhibit or stimulate biosynthetic processes. The data illustrating the lactobionic acid concentrations in cultures No. 4–7 were analysed statistically to find whether this interaction between pH (regulated or unregulated) and the form of bacteria (free or microencapsulated ones) may have occurred in our study. Table 1 shows the results of statistical analysis.

**Table 1:** Concentration of lactobionic acid during cultures nos. 4–7

Time (h)	Concentration of lactobionic acid (mg/cm^3^)			
	Culture 4	Culture 5	Culture 6 Free bacteria	Culture 7 microencapsulated bacteria
24	${5.80}^{\mathrm{c}1}$	${2.27}^{\mathrm{b}1}$	${0}^{\mathrm{a}1}$	${22.03}^{\mathrm{d}1}$
48	${5.58}^{\mathrm{b}2}$	${2.15}^{\mathrm{a}2}$	${0.22}^{\mathrm{a}2}$	${5.99}^{\mathrm{b}2}$
72	${6.50}^{\mathrm{b}3}$	${2.44}^{\mathrm{a}3}$	${0.23}^{\mathrm{a}3}$	${8.10}^{\mathrm{b}3}$
96	${8.54}^{\mathrm{c}4}$	${1.96}^{\mathrm{b}4}$	${0.028}^{\mathrm{a}4}$	${3.30}^{\mathrm{b}4}$
120	${3.13}^{\mathrm{b}5}$	${3.48}^{\mathrm{b}5}$	${0.14}^{\mathrm{a}5}$	${5.77}^{\mathrm{c}5}$
144	${6.08}^{\mathrm{c}6}$	${3.99}^{\mathrm{b}6}$	${0.15}^{\mathrm{a}6}$	${3.68}^{\mathrm{b}6}$
168	${7.96}^{\mathrm{c}7}$	${2.43}^{\mathrm{b}7}$	${0.33}^{\mathrm{a}7}$	${1.41}^{\mathrm{b}7}$
192	${8.43}^{\mathrm{c}8}$	${3.65}^{\mathrm{b}8}$	${0.85}^{\mathrm{a}8}$	${10.38}^{\mathrm{c}8}$
216	${4.59}^{\mathrm{c}9}$	${0.16}^{\mathrm{a}9}$	${1.47}^{\mathrm{b}9}$	${5.19}^{\mathrm{c}9}$
240	${7.82}^{\mathrm{b}10}$	${1.57}^{\mathrm{a}10}$	${1.47}^{\mathrm{a}10}$	${0.77}^{\mathrm{a}10}$
264	${6.14}^{\mathrm{b}11}$	${1.74}^{\mathrm{a}11}$	${1.23}^{\mathrm{a}11}$	${9.85}^{\mathrm{c}11}$
288	${8.03}^{\mathrm{c}12}$	${2.56}^{\mathrm{b}12}$	${1.13}^{\mathrm{a}12}$	${2.59}^{\mathrm{b}12}$
312	${3.57}^{\mathrm{b}13}$	${2.87}^{\mathrm{b}13}$	${1.16}^{\mathrm{a}13}$	${5.28}^{\mathrm{c}13}$

Indexes a1-a13, b1-b12, c1-c12 and d1 denote significant differences between concentrations of lactobionic acid in the same hours

The statistical analysis consisted in checking the homogeneity of variance at individual times of all cultures by means of Cochran’s, Hartley’s and Bartlett’s tests at a significance level α = 0.05. All the variances were homogeneous. Next, the hypothesis that pH (regulated and unregulated) and the form of bacteria (free and microencapsulated ones) had no influence on the yield of lactobionic acid was rejected after the classic analysis of variance. The analyses showed that the joint use of microencapsulated *P. taetrolens* bacteria and pH positively affected the efficiency of biosynthesis of lactobionic acid from whey.

The microscopic images of specimens from all the cultures with microencapsulated bacteria and bacteria without capsules were very similar. There was no foreign microflora in any of the cultures.

As there are few publications on the microbial LBA biosynthesis from whey, it is difficult to evaluate the results of our study. In similar studies there was higher effect of the bioconversion of lactose to LBA. Alonso et al. (2011) observed that after 32 h with the 30% inoculum the LBA concentration was 42.4 g/dm^3^. The same authors noted 90% in a later study (Alonso et al. 2013). These findings point to the high potential of this method, which has not been fully investigated yet. It might be helpful to conduct research optimising environmental conditions such as pH, temperature, medium flow rate and dissolved oxygen concentration. Genetic factors, especially the introduction and expression of adequate genes, should also be taken into consideration [11].

Analyses of microscopic images showed that cell morphology of *Pseudomonas taetrolens* did not change during the production of lactobionic acid. The cell counts of *Pseudomonas taetrolens* in the course of lactobionic acid production using encapsulated bacteria were very similar up to 120 h of culture. Moreover, the count of live cells of encapsulated *Pseudomonas taetrolens* was also determined during oxidation of lactose to lactobionic acid. The culture was obtained from the inoculum after 24 h incubation at a temperature of 30˚C, from stationary culture run in a bioreactor after 24 h of culture, as well as continuous culture run in a bioreactor after 24 h of culture. Obtained results indicate a tenfold increase in cell counts in capsules in the culture run in a bioreactor in comparison to the stationary culture, thus in order to optimise production of lactobionic acid cultures in a bioreactor were run under controlled and monitored conditions.

During bioconversion of lactose using bacteria *Pseudomonas taetrolens* DSM 21104 temperature, time, aeration and pH nave an effect on the yield of lactobionic acid*.* An experiment conducted by Alonso et al. (2011) [28] showed oxidative capacity of *P. teatrolens* in relation to lactose and its dependence on the presence of oxygen. For this purpose new aeration and mixing conditions were introduced in the bioreactor. The lactobionic acid titre is obtained by the application of oxygen deficit at the growth phase. This process shows a very low bioconversion efficiency of 37%, leaving 26.5 g/l lactose after 60 h. These results show that the limited inflow of oxygen markedly inhibits the reaction of oxidation of lactose to lactobionic acid. In the experiments parameters which were adopted in all cultures were as aeration – 0.5 dm^3^/min and mixing—120 rpm.

The effect of different oxygen concentrations on bioprocess parameters and production of lactobionic acid from whey was tested. Lower aeration levels contributed to an increased growth of *P. taetrolens*, the highest maximum concentration of biomass at 1.36 g/l is obtained for 0.5 l/min. Similar values of maximum biomass concentration at 1.25 and 1.22 g/l were reached at 1 and 1.5 l/min of aeration rate. In turn, the application of the highest aeration rate 2 l/min provides only 1.16 g/l biomass. When cells are not exposed to aeration a markedly limited cell growth to 0.057 g/l is observed [28].

Oxidation of lactobionic acid by *P. taetrolens* was investigated at various mixing values at a constant mixing velocity. A higher mixing frequency stimulates cell growth in *Pseudomonas taetrolens*, the maximum biomass of 1.06, 1.25 1.29, 1.40 and 1.48 g/l is obtained at 150, 350, 500, 700 and 1000 rpm, respectively. However, the best results in the production of lactobionic acid were recorded in the mixing process at 350 rpm with the yield of 0.7 g/l already after 10 h fermentation.

An experiment was conducted by Alonso et al. (2011) [28] using *Pseudomonas taetrolens.* The logarithmic phase using the inoculum phase at the stationary growth phase increased considerably, while the lack of the delay phase was observed for 12 h culture. Based on the recorded results we may see that the use of a bacterial culture with an extended incubation time led to an increase in the delay phase as well as a reduced yield of lactobionic acid production.

An experiment was conducted by Alonso et al. (2011) [28] using *Pseudomonas taetrolens.* A high inoculum density promotes rapid activation of *P. taetrolens* cells. At a 30% inoculation we obtain maximum cell density of 1.35 g/l. Process parameters were tested at 5, 20 and 30% inoculation levels, corresponding to 0.09, 0.36 and 0.56 g/l biomass. The application of greater numbers of microorganisms (e.g. when comparing inoculation at 5% and 30%) guarantees a shortening of growth time from 10 to 4 h. We may also observe a considerable reduction of oxidation time from 72 h at 5% inoculation to 32 h at 30% inoculation of the medium. Maximum volume efficiency of lactobionic acid production was obtained using the highest concentration of the inoculum (30%), yielding 1.12 g/lh in comparison to 5%, where 0.59 g/lh lactobionic acid is obtained. We may also observe changes in pH, which—as shown by the recorded results—decreases with an increase in the concentration of the inoculum. Alginate microcapsules serve a protective function in relation to bacteria *Pseudomonas taetrolens* DSM 21104 and yields of lactobionic acid obtained from whey using encapsulated microorganisms are higher at pH maintained at 6.25 during bioreactor culture.

In study of Patel et al. (2018) [29], biological methanol production under repeated batch conditions by immobilized *Methylocystis bryophila*, using simulated biogas of methane (CH_4_) and carbon dioxide (CO_2_) as a feed is demonstrated for the first time. In this study, biological methanol production under repeated batch conditions by immobilized *Methylocystis bryophila*, using simulated biogas of methane (CH_4_) and carbon dioxide (CO_2_) as a feed is demonstrated for the first time. Methanol yield was increased using covalently immobilized cells and the simulated gas mixture CH_4_:CO_2_:H_2_ (6:3:2 ratio). In the study of Patel et al. (2021) [30] lignocellulosic biowastes, which are rich in sugars, be used as low-cost feed to produce environmentally friendly fuels such as hydrogen (H_2_). The continuous production of H_2_ from poplar biomass using well-defined mixed cultures in immobilized state under non-sterile conditions. This approach may enable to meet the increasing demand for cleaner energy-rich fuels on a large-scale in a steady manner.

Concentration of lactobionic acid obtained using microencapsulated bacteria *Pseudomonas taetrolens* DSM 21104 is greater than using non-encapsulated bacteria. Obtained results indicate a tenfold increase in cell counts in capsules in the culture run in a bioreactor in comparison to the stationary culture, thus in order to optimise production of lactobionic acid cultures in a bioreactor were run under controlled and monitored conditions. The highest concentration of lactobionic acid was obtained at 24 h of culture and it amounted to 22.03 mg/cm^3^. Experiments confirmed that alginate capsules were permeable for enzymes oxidising lactose to lactobionic acid, produced extracellularly by *Pseudomonas taetrolens.*

Ambient pH has a significant effect on the production of lactobionic acid and it should be maintained at 6.25 – 6.5. The content of lactose in the course of culture should decrease due to the enzymatic oxidation of tis sugar to glucono-δ–lactone and then to lactobionic acid. The reduction of lactose concentration is not always accompanied by an increase in the concentration of lactobionic acid, which is related with the formation of by-products such as acetic, propionic, butyric and isobutyric acids. Conducted analyses showed that the structure of microcapsules was deformed and microorganisms penetrated from capsules to the medium, as evidenced by their presence in the liquid culture medium. The structure of calcium alginate was weakened by sodium ions and lactobionic acid present in the culture and the condensation of biomass at capsule membranes caused their degradation.

The production of lactobionic acid using bacteria *Pseudomonas taetrolens* DSM 21104 on whey used as the medium proved to be more efficient when applying encapsulated bacteria rather than microorganisms with no encapsulation. An additional advantage of encapsulation is connected with easy separation of microbial cells from the culture medium and it may be utilised in the future. Utilisation of a by-product of the dairy industry is an additional advantage of this process and it expands the scope of whey management. pH and the use of *P. taetrolens* encapsulated in microcapsules jointly beneficial affected the efficiency of lactobionic acid biosynthesis from whey.

## Supplementary Information

Below is the link to the electronic supplementary material.

Fig. S1 The encapsulated bacteria in alginate capsule. Fig. S2. Concentration of lactobionic acid and lactose during cultures no. 1 [mg/cm^3^]. Fig. S3. Concentration of lactobionic acid and lactose during cultures no. 2 [mg/cm^3^]. Fig. S4. Concentration of lactobionic acid and lactose during cultures no. 3 [mg/cm^3^]. Fig. S5. Concentration of lactobionic acid and lactose during cultures no. 4 [mg/cm^3^]. Fig. S6. Concentration of lactobionic acid and lactose during cultures no. 5 [mg/cm^3^]. Fig. S7. Concentration of lactobionic acid and lactose during cultures no. 6 [mg/cm^3^]. Fig. S8. Concentration of lactobionic acid and lactose during cultures no. 7 [mg/cm^3^]

## Data Availability

All data generated or analysed during this study are included in this published article (and its supplementary information files).
