# Supplementary figures and images for: Biosynthesis of Lactobionic Acid in Whey-Containing Medium by Microencapsulated and Free Bacteria of Pseudomonas taetrolens
Source: Indian J Microbiol. 2021 May 11;61(3):315–23. doi: 10.1007/s12088-021-00944-4 (PMC8263841; doi:10.1007/s12088-021-00944-4)

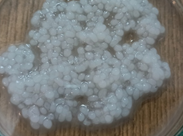


Fig. S1


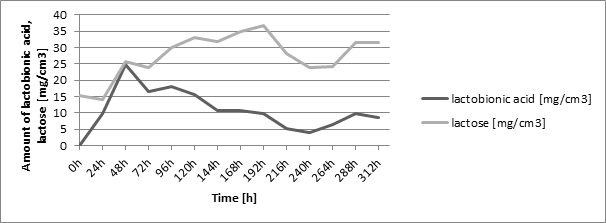


Fig. S1


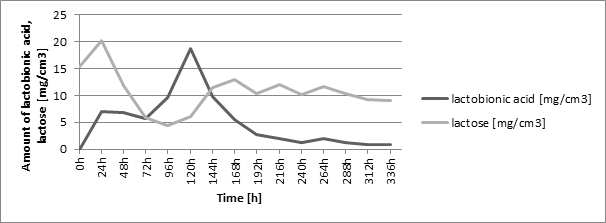


Fig. S2


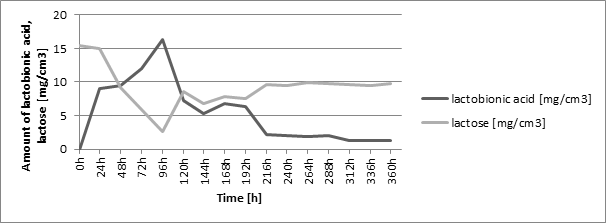


Fig. S3


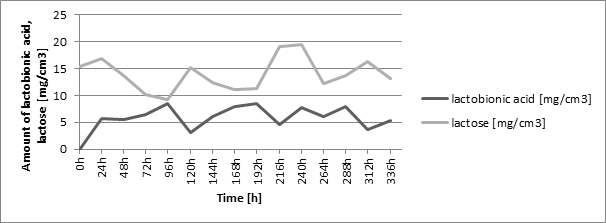


Fig. S4


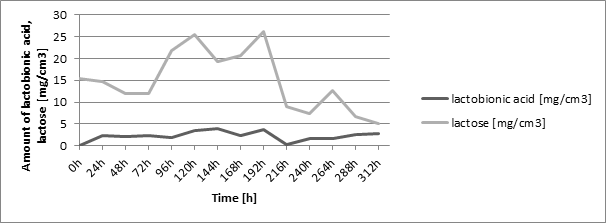


Fig. S5


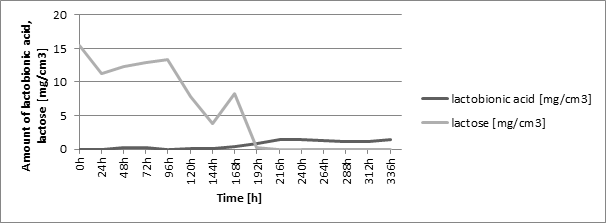


Fig. S6


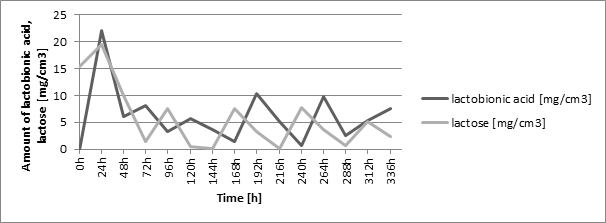


Fig. S7

Supplement: Supplementary file 1 — Fig. S1 The encapsulated bacteria in alginate capsule. Fig. S2. Concentration of lactobionic acid and lactose during cultures no. 1 [mg/cm3]. Fig. S3. Concentration of lactobionic acid and lactose during cultures no. 2 [mg/cm3]. Fig. S4. Concentration of lactobionic acid and lactose during cultures no. 3 [mg/cm3]. Fig. S5. Concentration of lactobionic acid and lactose during cultures no. 4 [mg/cm3]. Fig. S6. Concentration of lactobionic acid and lactose during cultures no. 5 [mg/cm3]. Fig. S7. Concentration of lactobionic acid and lactose during cultures no. 6 [mg/cm3]. Fig. S8. Concentration of lactobionic acid and lactose during cultures no. 7 [mg/cm3] [file 12088_2021_944_MOESM1_ESM.docx]
